# Supplementary material for: Characterization of BV6-Induced Sensitization to the NK Cell Killing of Pediatric Rhabdomyosarcoma Spheroids
Source: Cells. 2023 Mar 15;12(6):906. doi: 10.3390/cells12060906 (PMC10047333; doi:10.3390/cells12060906)
Supplement: Supplementary file 1 [file cells-12-00906-s001.zip › cells-2205629-supplementary.pptx]

## Slide 1
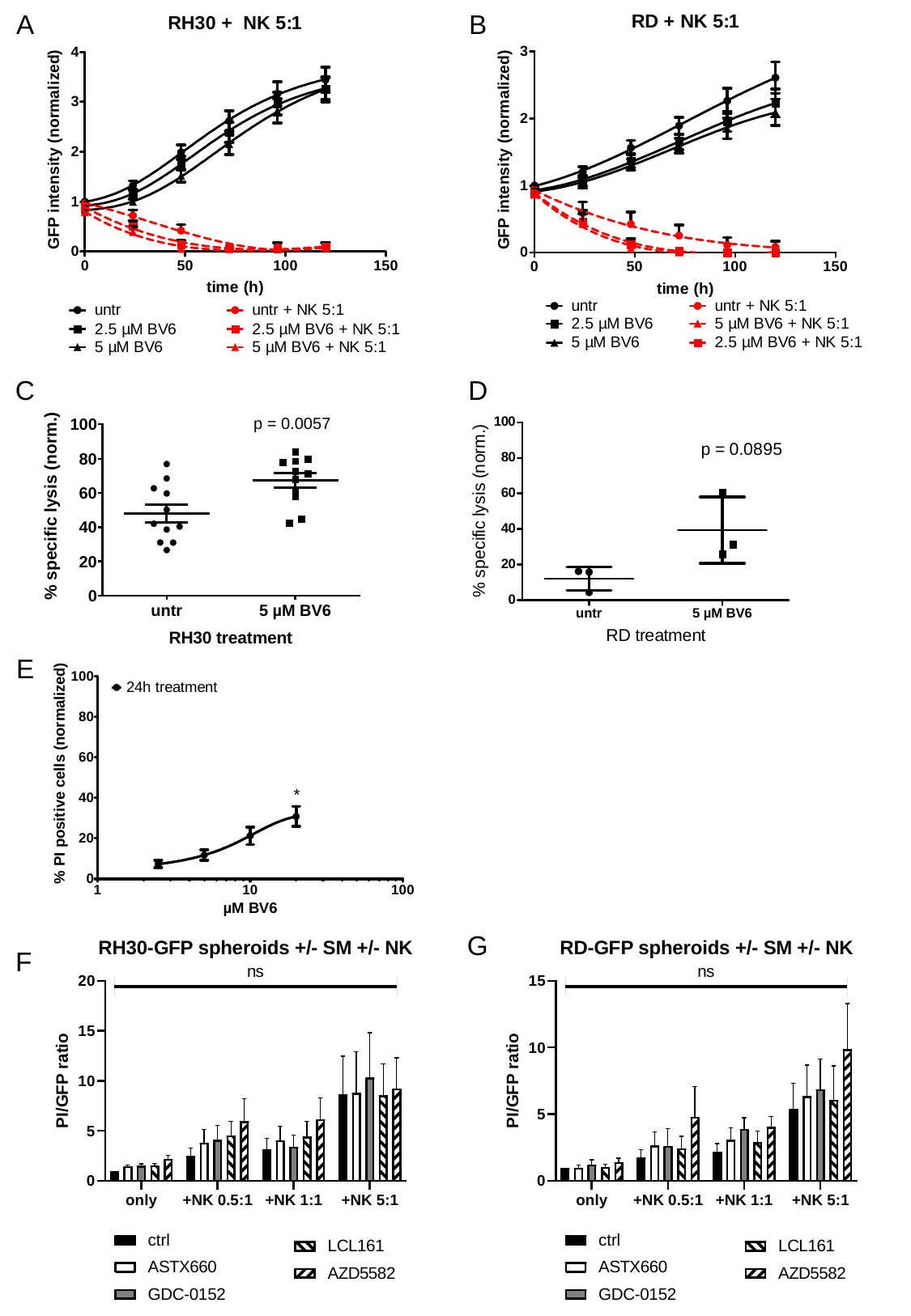

A
B
C
D
E
G
F

## Slide 2
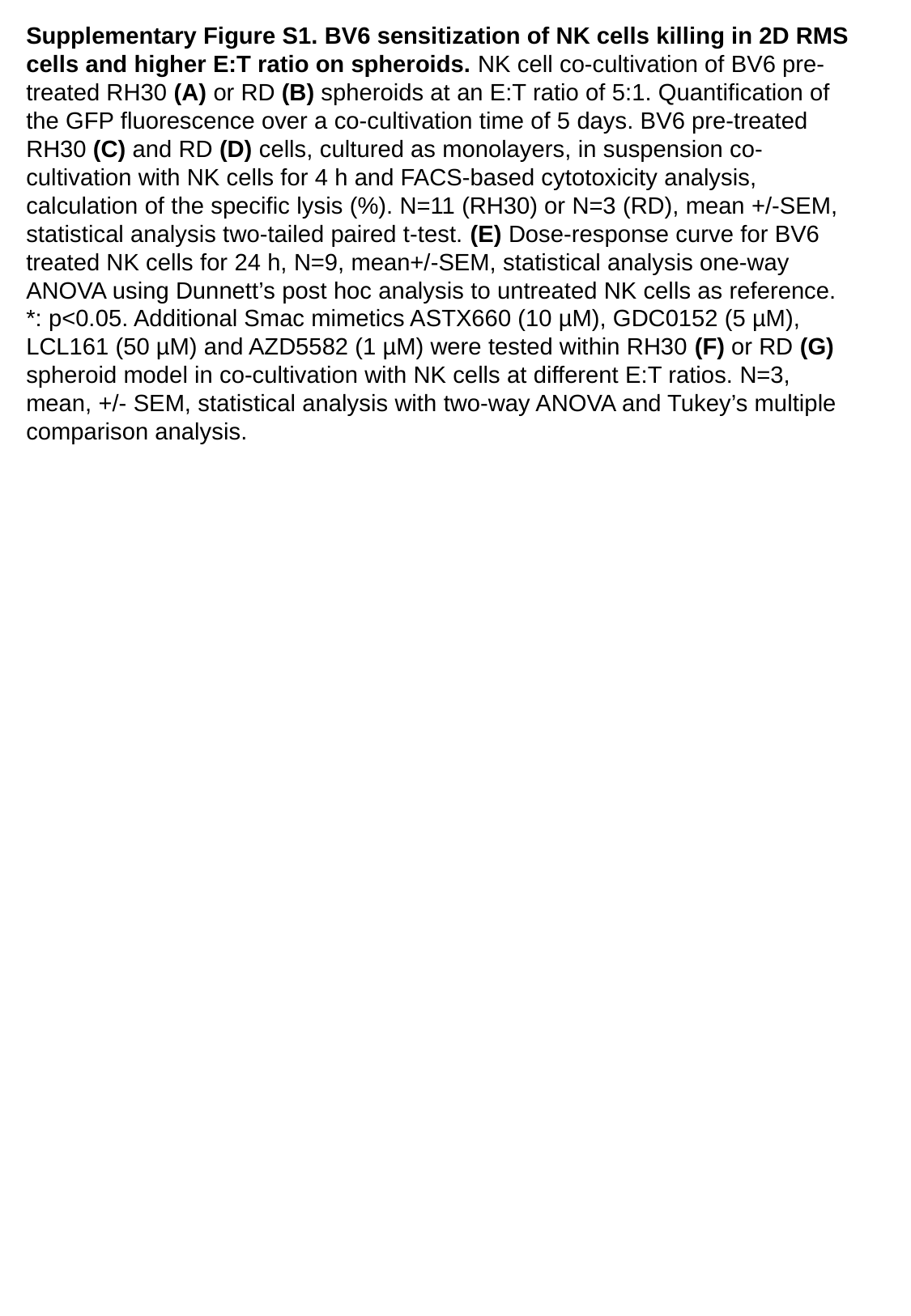

Supplementary Figure S1. BV6 sensitization of NK cells killing in 2D RMS cells and higher E:T ratio on spheroids. NK cell co-cultivation of BV6 pre-treated RH30 (A) or RD (B) spheroids at an E:T ratio of 5:1. Quantification of the GFP fluorescence over a co-cultivation time of 5 days. BV6 pre-treated RH30 (C) and RD (D) cells, cultured as monolayers, in suspension co-cultivation with NK cells for 4 h and FACS-based cytotoxicity analysis, calculation of the specific lysis (%). N=11 (RH30) or N=3 (RD), mean +/-SEM, statistical analysis two-tailed paired t-test. (E) Dose-response curve for BV6 treated NK cells for 24 h, N=9, mean+/-SEM, statistical analysis one-way ANOVA using Dunnett’s post hoc analysis to untreated NK cells as reference. *: p<0.05. Additional Smac mimetics ASTX660 (10 µM), GDC0152 (5 µM), LCL161 (50 µM) and AZD5582 (1 µM) were tested within RH30 (F) or RD (G) spheroid model in co-cultivation with NK cells at different E:T ratios. N=3, mean, +/- SEM, statistical analysis with two-way ANOVA and Tukey’s multiple comparison analysis.

## Slide 3
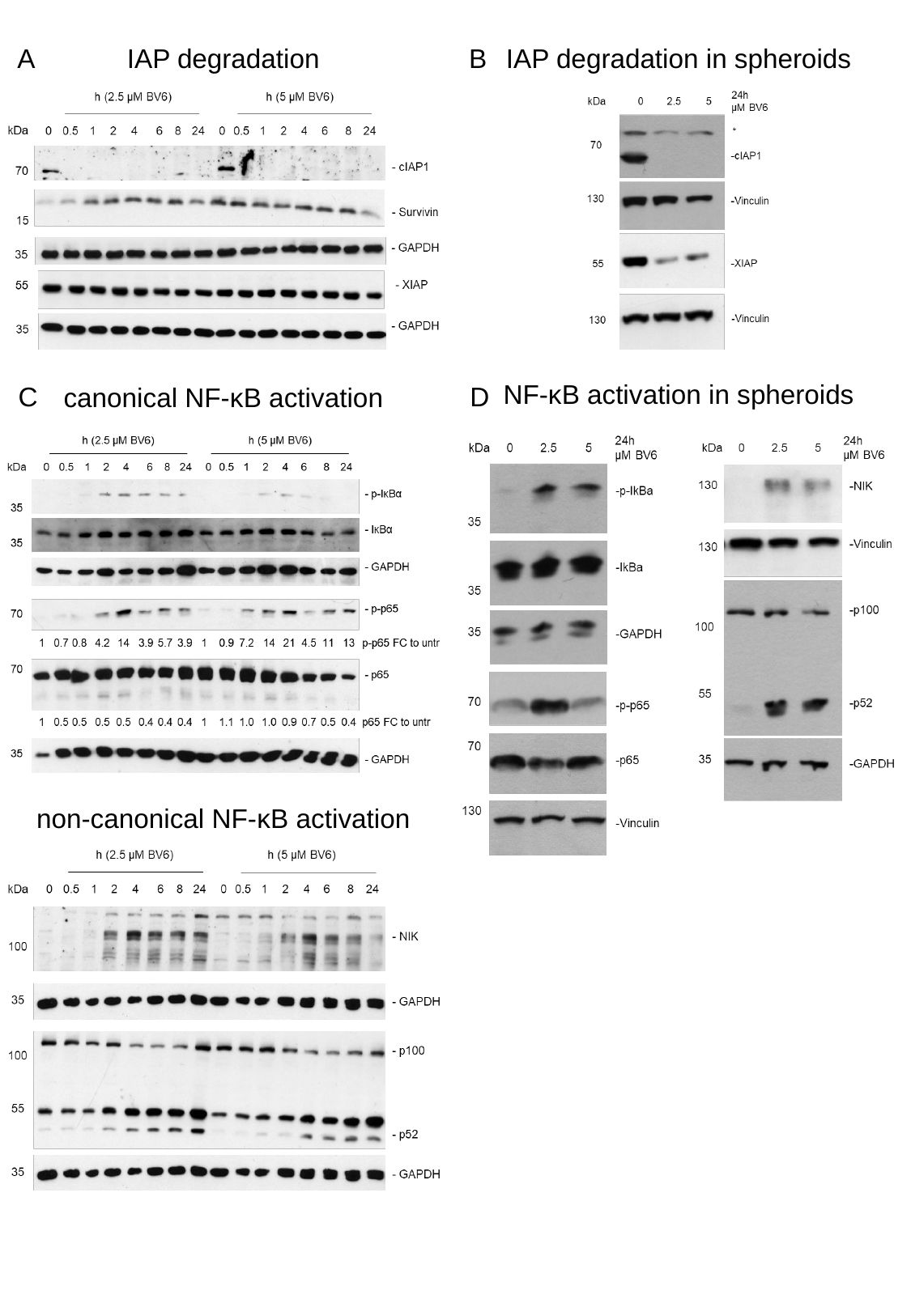

A
IAP degradation
B
IAP degradation in spheroids
NF-κB activation in spheroids
C
D
canonical NF-κB activation
non-canonical NF-κB activation

## Slide 4
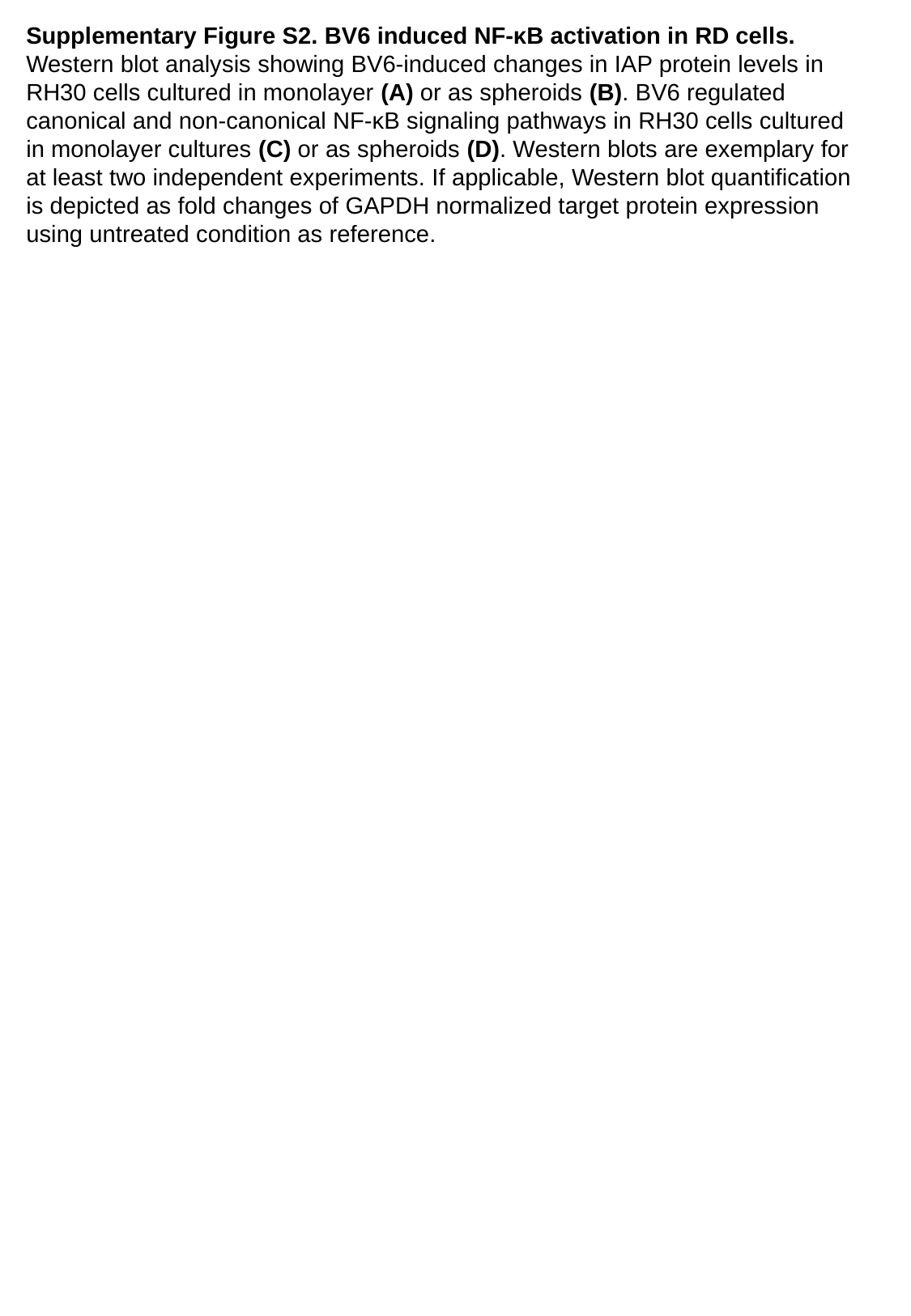

Supplementary Figure S2. BV6 induced NF-κB activation in RD cells. Western blot analysis showing BV6-induced changes in IAP protein levels in RH30 cells cultured in monolayer (A) or as spheroids (B). BV6 regulated canonical and non-canonical NF-κB signaling pathways in RH30 cells cultured in monolayer cultures (C) or as spheroids (D). Western blots are exemplary for at least two independent experiments. If applicable, Western blot quantification is depicted as fold changes of GAPDH normalized target protein expression using untreated condition as reference.

## Slide 5
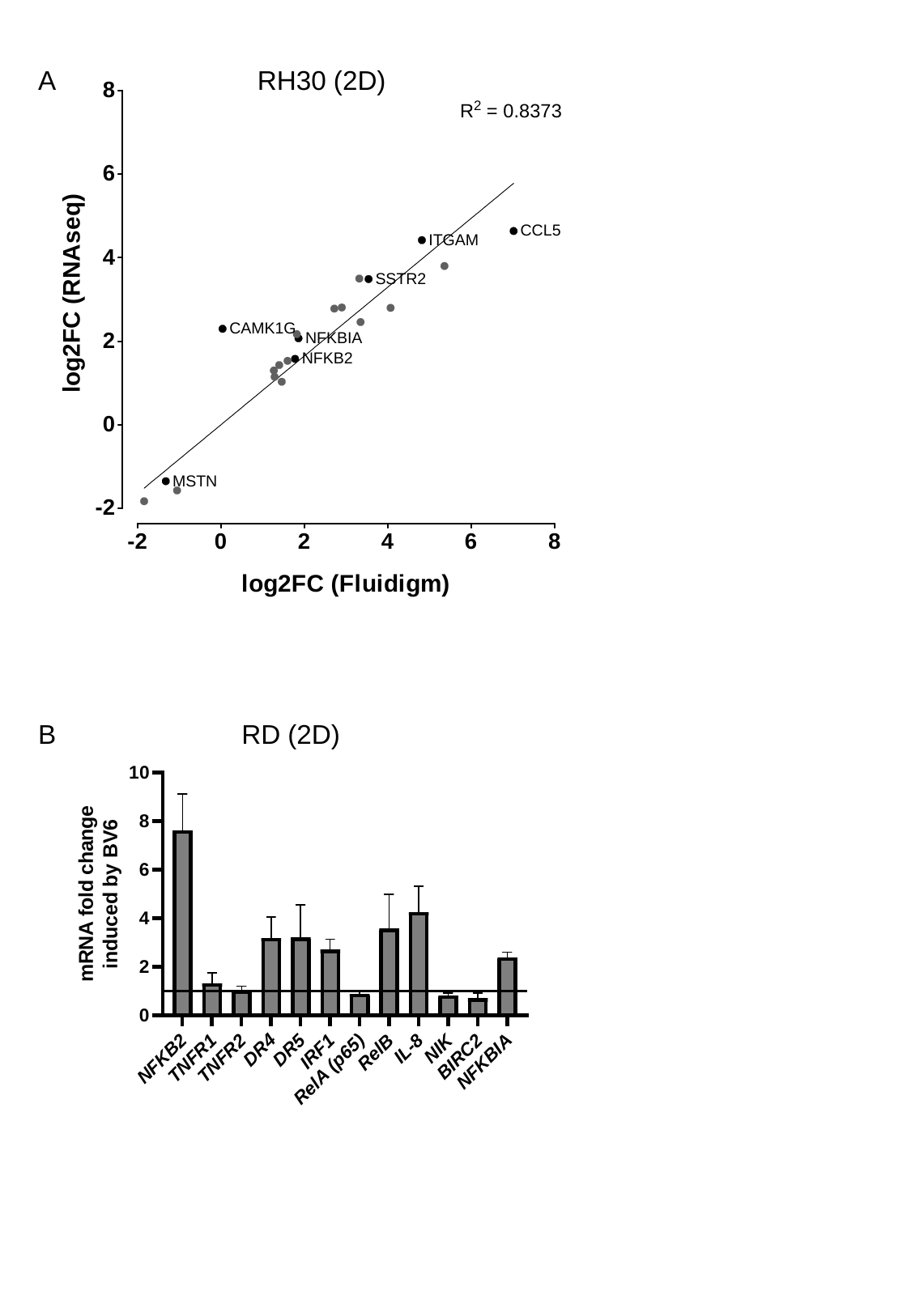

A
RH30 (2D)
B
RD (2D)

## Slide 6
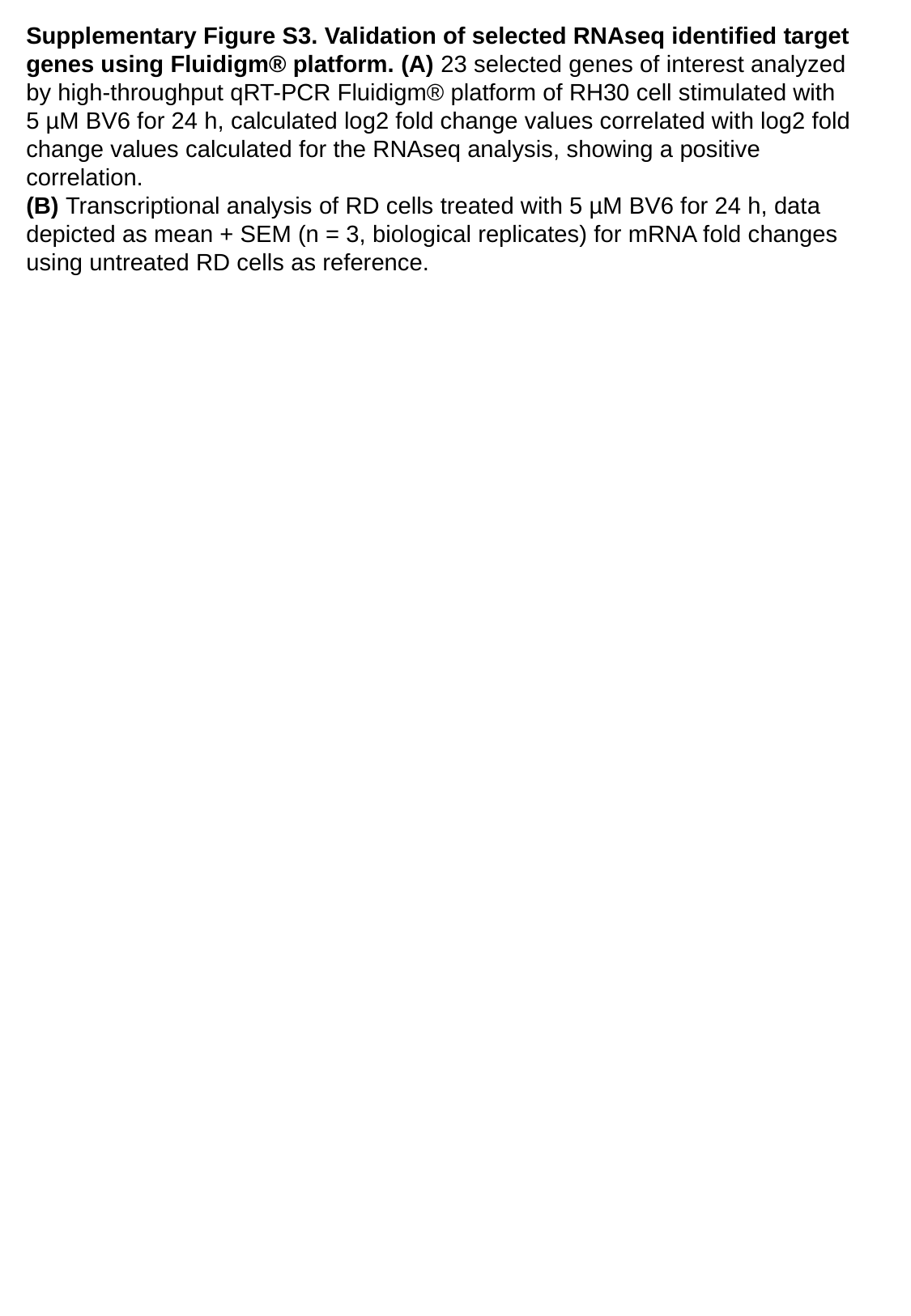

Supplementary Figure S3. Validation of selected RNAseq identified target genes using Fluidigm® platform. (A) 23 selected genes of interest analyzed by high-throughput qRT-PCR Fluidigm® platform of RH30 cell stimulated with 5 µM BV6 for 24 h, calculated log2 fold change values correlated with log2 fold change values calculated for the RNAseq analysis, showing a positive correlation.
(B) Transcriptional analysis of RD cells treated with 5 µM BV6 for 24 h, data depicted as mean + SEM (n = 3, biological replicates) for mRNA fold changes using untreated RD cells as reference.

## Slide 7
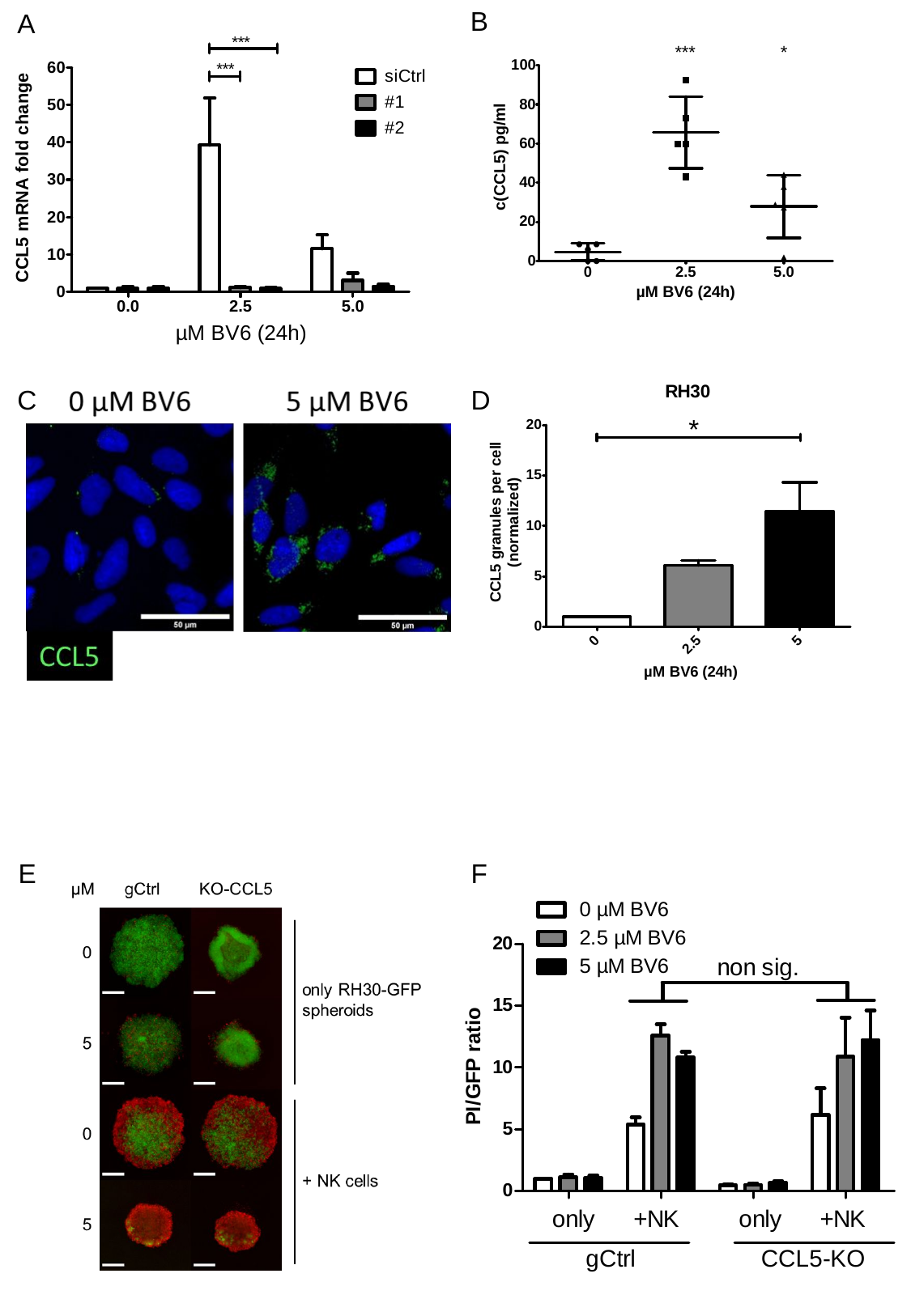

B
A
C
D
E
F

## Slide 8
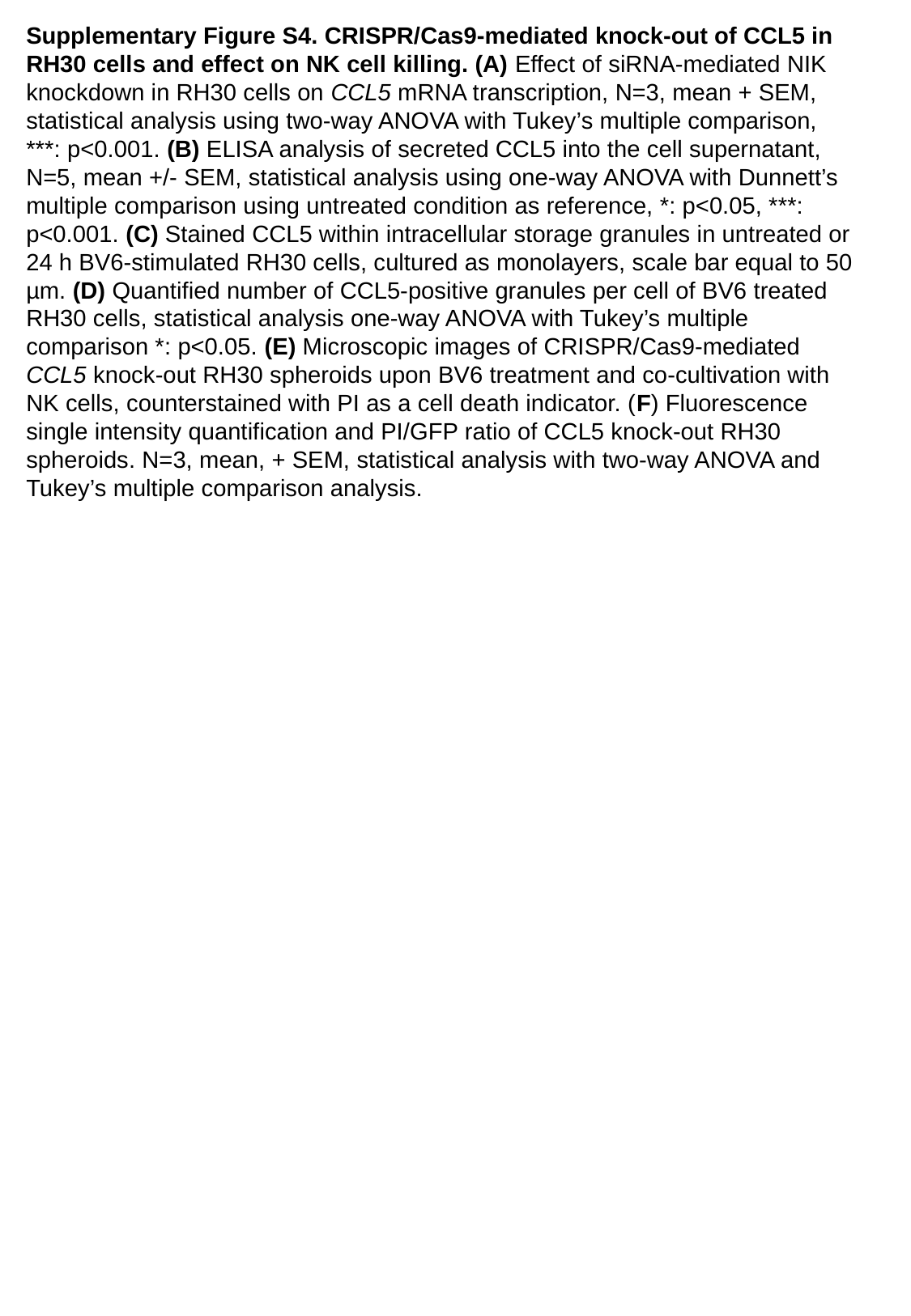

Supplementary Figure S4. CRISPR/Cas9-mediated knock-out of CCL5 in RH30 cells and effect on NK cell killing. (A) Effect of siRNA-mediated NIK knockdown in RH30 cells on CCL5 mRNA transcription, N=3, mean + SEM, statistical analysis using two-way ANOVA with Tukey’s multiple comparison, ***: p<0.001. (B) ELISA analysis of secreted CCL5 into the cell supernatant, N=5, mean +/- SEM, statistical analysis using one-way ANOVA with Dunnett’s multiple comparison using untreated condition as reference, *: p<0.05, ***: p<0.001. (C) Stained CCL5 within intracellular storage granules in untreated or 24 h BV6-stimulated RH30 cells, cultured as monolayers, scale bar equal to 50 µm. (D) Quantified number of CCL5-positive granules per cell of BV6 treated RH30 cells, statistical analysis one-way ANOVA with Tukey’s multiple comparison *: p<0.05. (E) Microscopic images of CRISPR/Cas9-mediated CCL5 knock-out RH30 spheroids upon BV6 treatment and co-cultivation with NK cells, counterstained with PI as a cell death indicator. (F) Fluorescence single intensity quantification and PI/GFP ratio of CCL5 knock-out RH30 spheroids. N=3, mean, + SEM, statistical analysis with two-way ANOVA and Tukey’s multiple comparison analysis.

## Slide 9
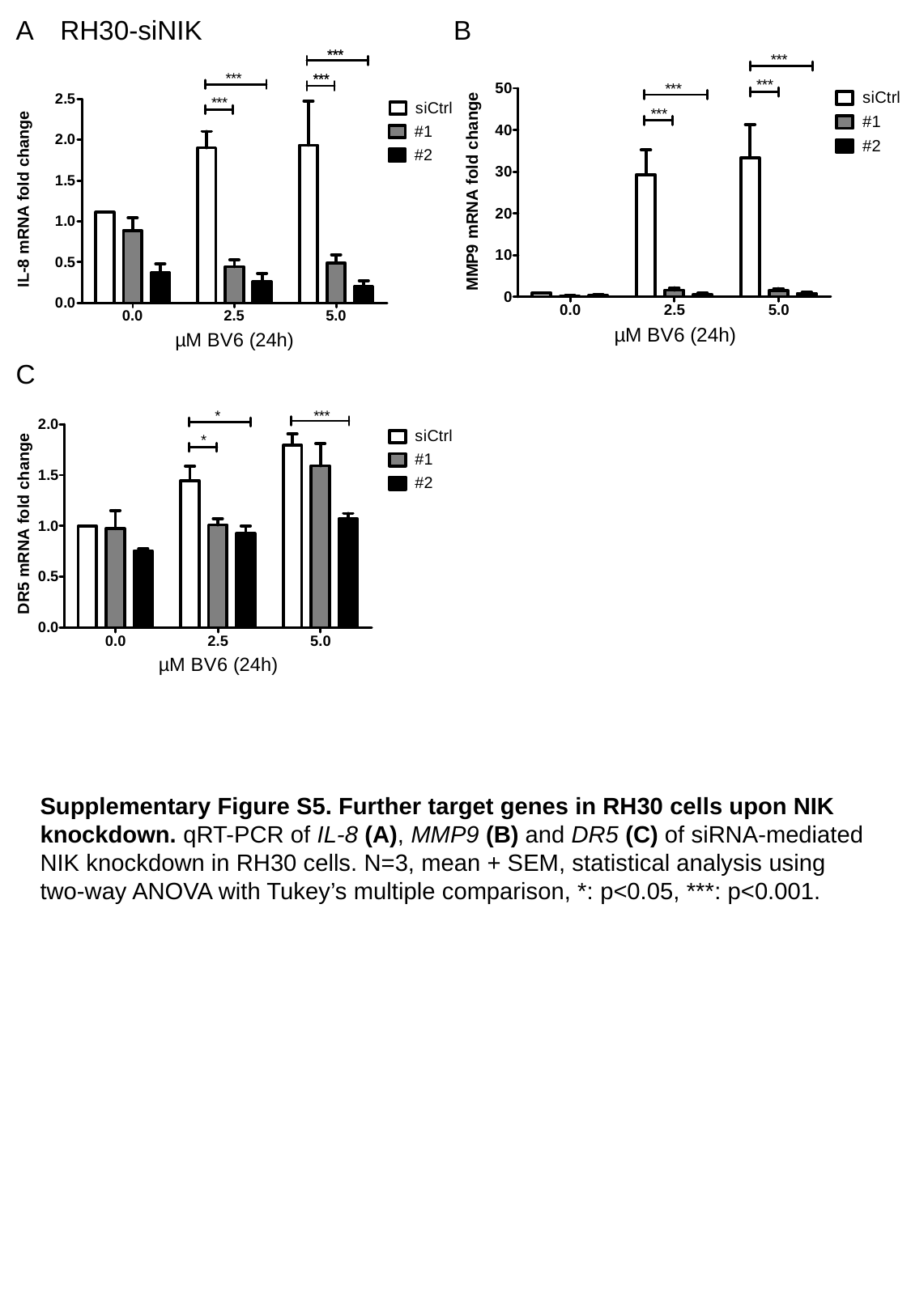

A
RH30-siNIK
B
C
Supplementary Figure S5. Further target genes in RH30 cells upon NIK knockdown. qRT-PCR of IL-8 (A), MMP9 (B) and DR5 (C) of siRNA-mediated NIK knockdown in RH30 cells. N=3, mean + SEM, statistical analysis using two-way ANOVA with Tukey’s multiple comparison, *: p<0.05, ***: p<0.001.

## Slide 10
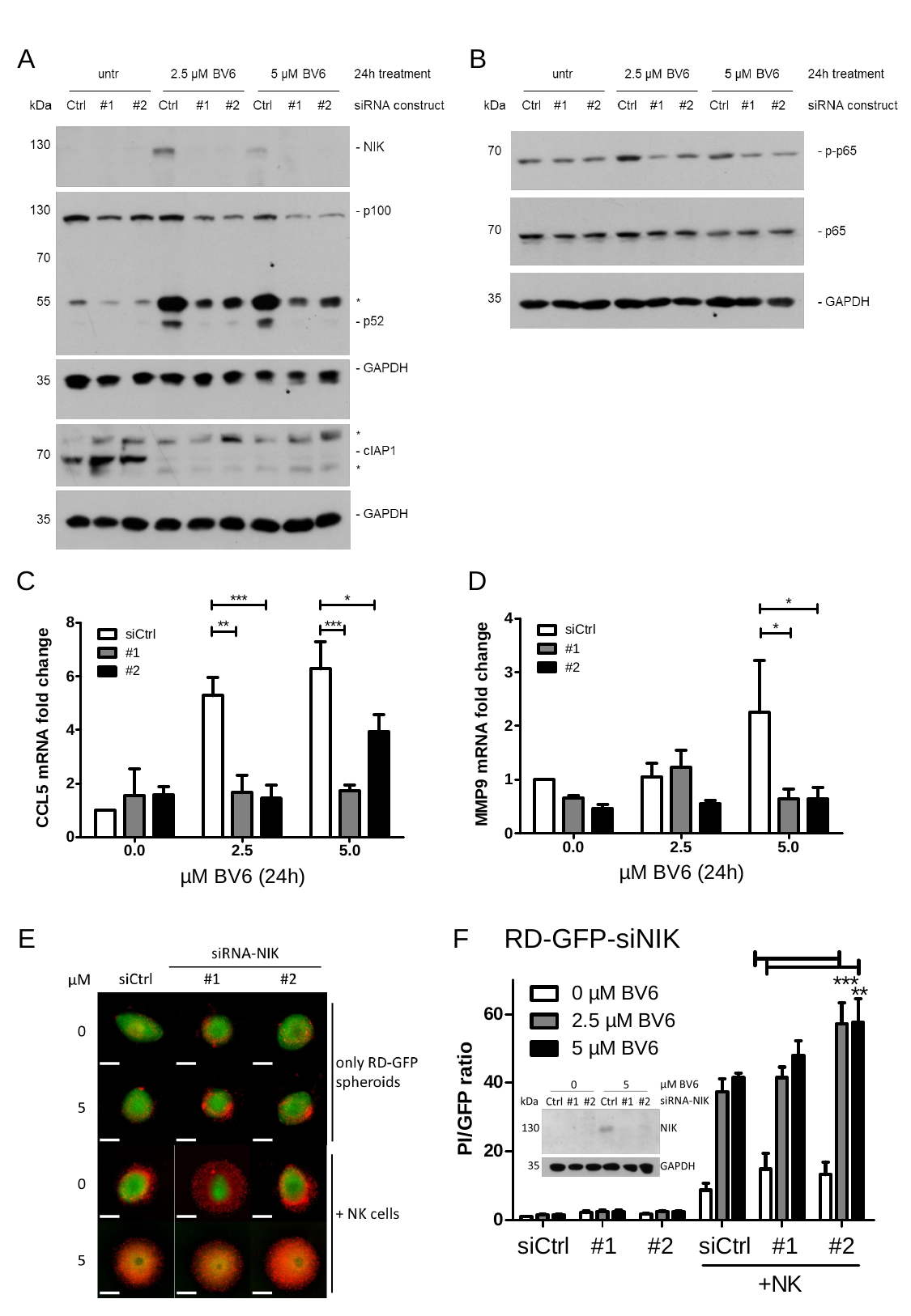

A
B
C
D
E
F
RD-GFP-siNIK

## Slide 11
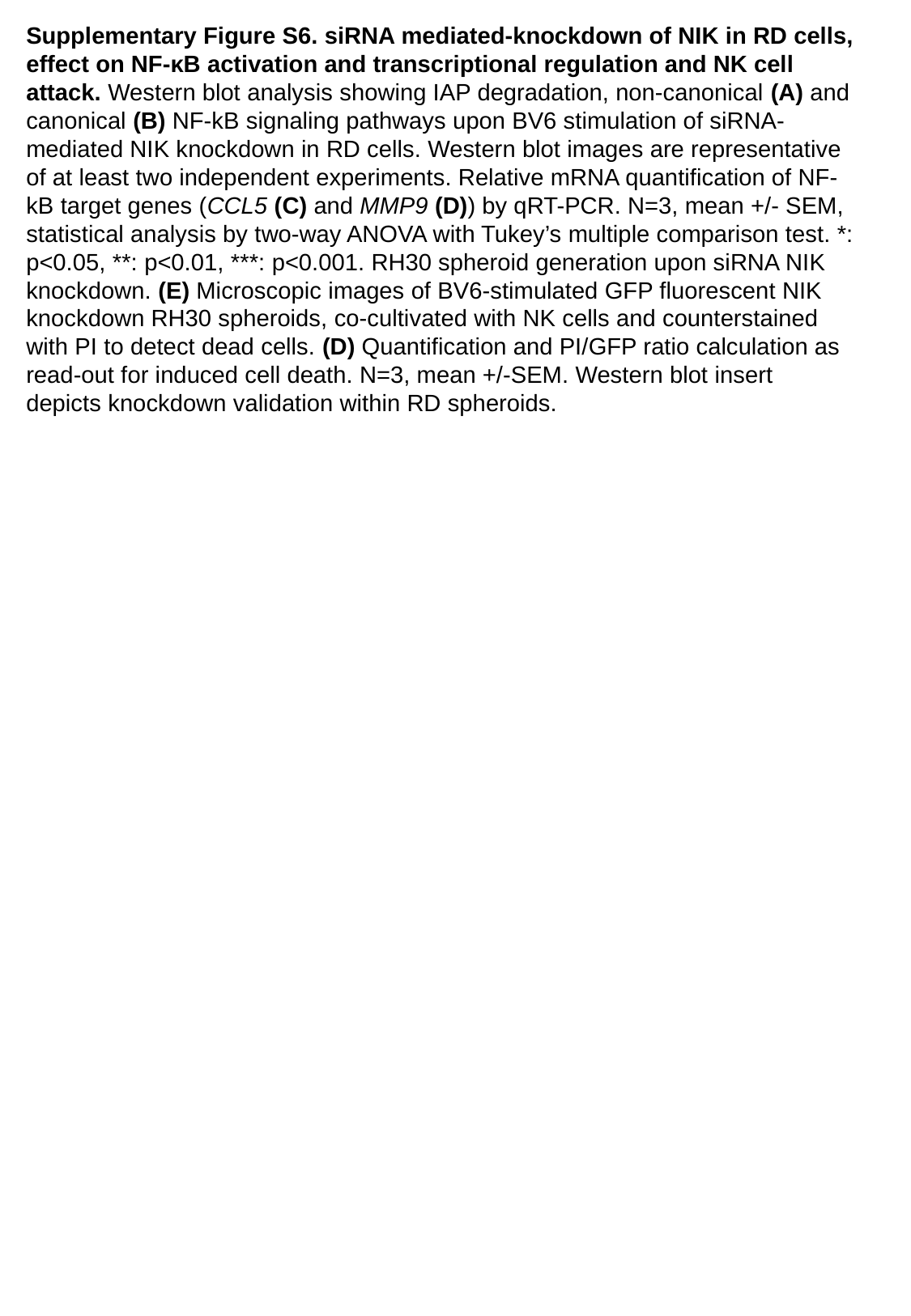

Supplementary Figure S6. siRNA mediated-knockdown of NIK in RD cells, effect on NF-κB activation and transcriptional regulation and NK cell attack. Western blot analysis showing IAP degradation, non-canonical (A) and canonical (B) NF-kB signaling pathways upon BV6 stimulation of siRNA-mediated NIK knockdown in RD cells. Western blot images are representative of at least two independent experiments. Relative mRNA quantification of NF-kB target genes (CCL5 (C) and MMP9 (D)) by qRT-PCR. N=3, mean +/- SEM, statistical analysis by two-way ANOVA with Tukey’s multiple comparison test. *: p<0.05, **: p<0.01, ***: p<0.001. RH30 spheroid generation upon siRNA NIK knockdown. (E) Microscopic images of BV6-stimulated GFP fluorescent NIK knockdown RH30 spheroids, co-cultivated with NK cells and counterstained with PI to detect dead cells. (D) Quantification and PI/GFP ratio calculation as read-out for induced cell death. N=3, mean +/-SEM. Western blot insert depicts knockdown validation within RD spheroids.

## Slide 12
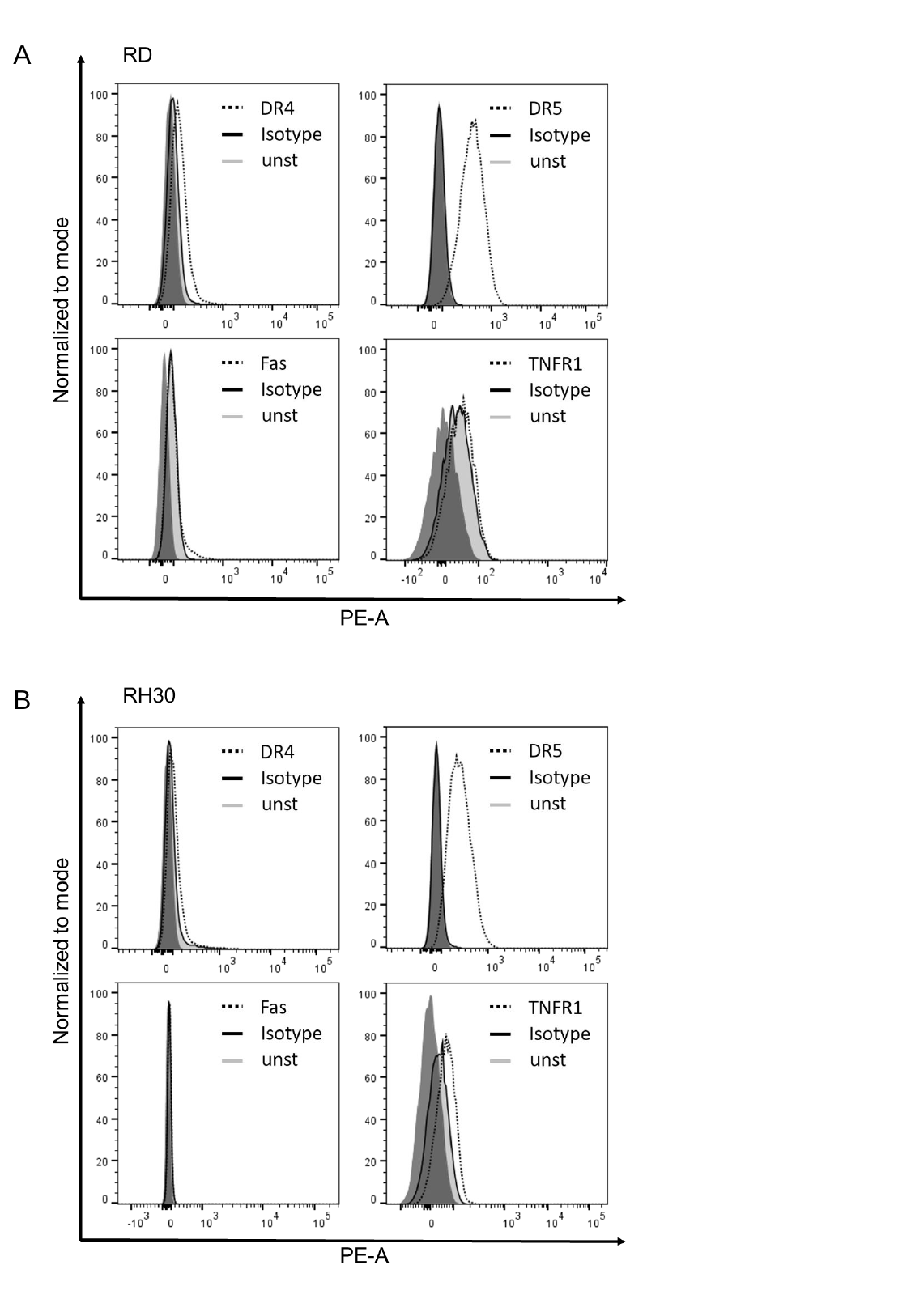

A
B

## Slide 13
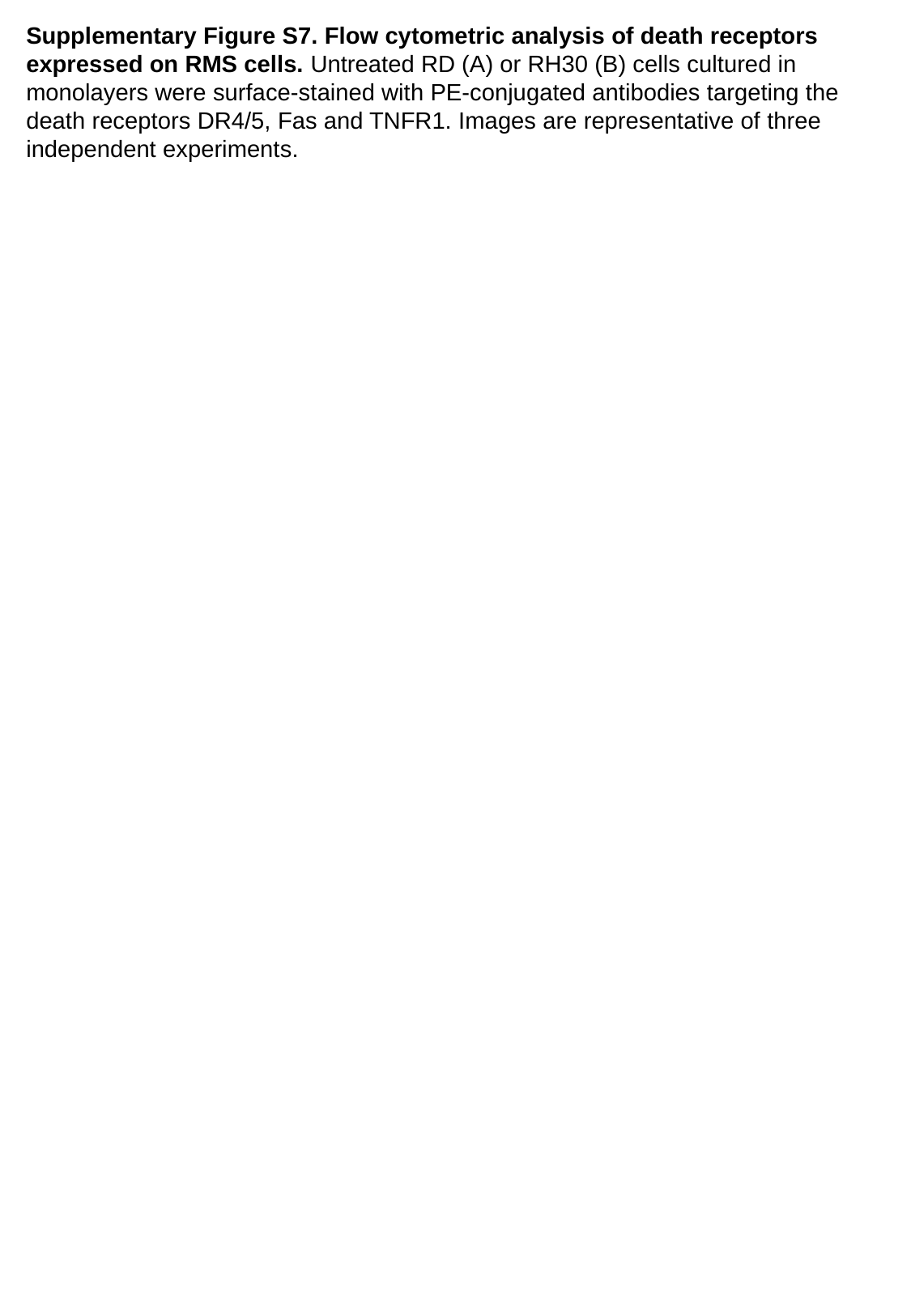

Supplementary Figure S7. Flow cytometric analysis of death receptors expressed on RMS cells. Untreated RD (A) or RH30 (B) cells cultured in monolayers were surface-stained with PE-conjugated antibodies targeting the death receptors DR4/5, Fas and TNFR1. Images are representative of three independent experiments.

## Slide 14
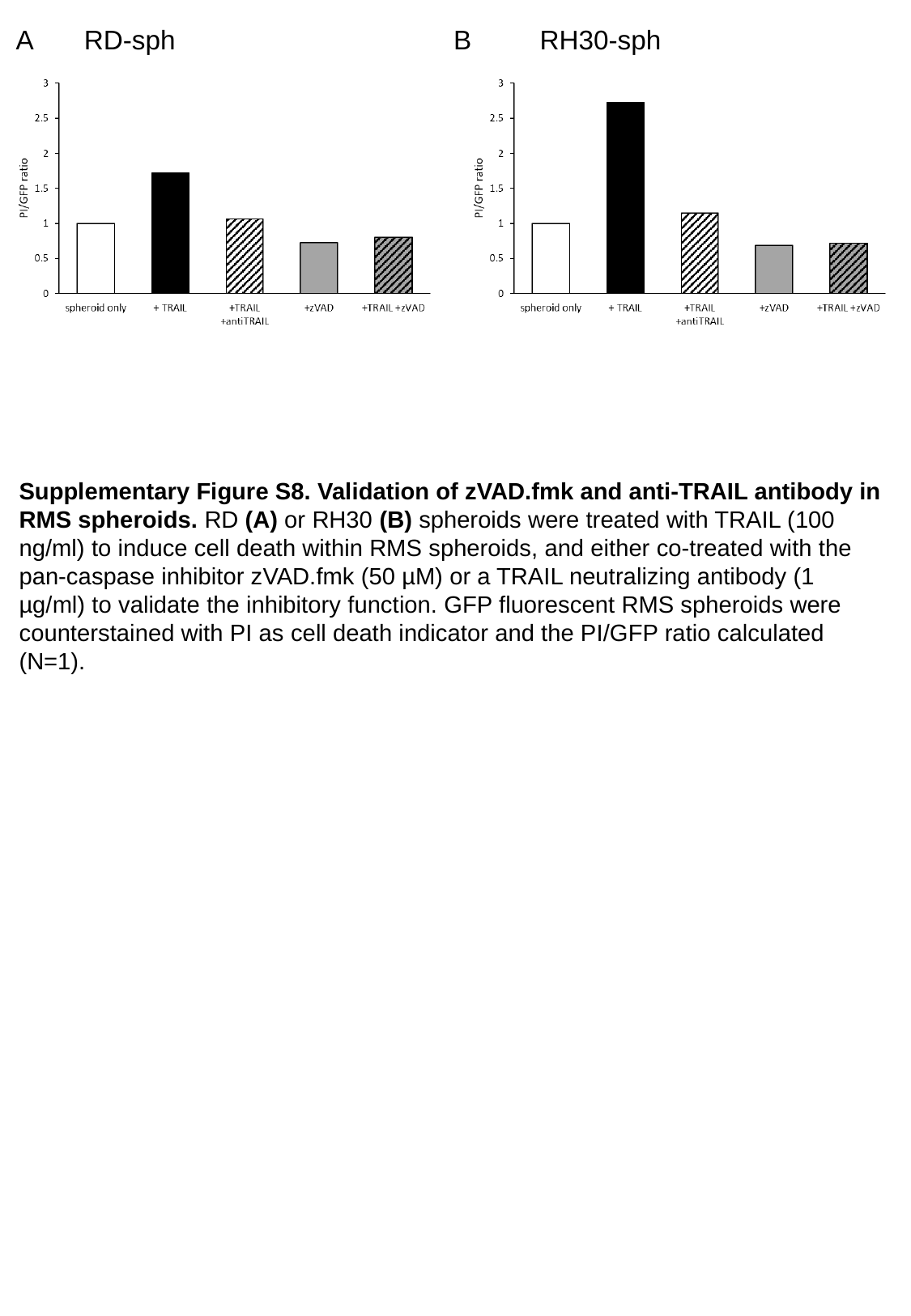

A
RD-sph
B
RH30-sph
Supplementary Figure S8. Validation of zVAD.fmk and anti-TRAIL antibody in RMS spheroids. RD (A) or RH30 (B) spheroids were treated with TRAIL (100 ng/ml) to induce cell death within RMS spheroids, and either co-treated with the pan-caspase inhibitor zVAD.fmk (50 µM) or a TRAIL neutralizing antibody (1 µg/ml) to validate the inhibitory function. GFP fluorescent RMS spheroids were counterstained with PI as cell death indicator and the PI/GFP ratio calculated (N=1).

## Slide 15
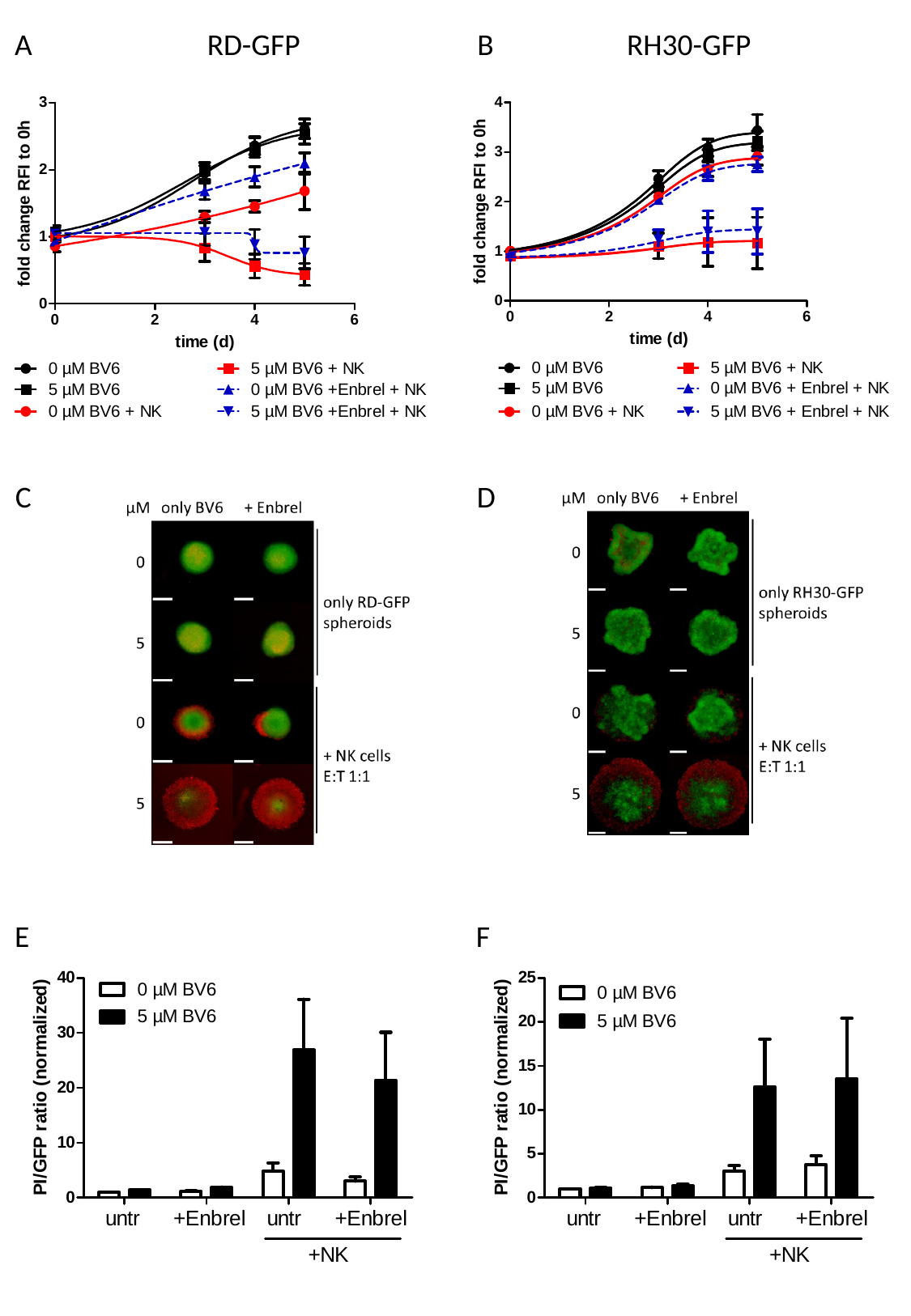

A
RD-GFP
B
RH30-GFP
C
D
E
F

## Slide 16
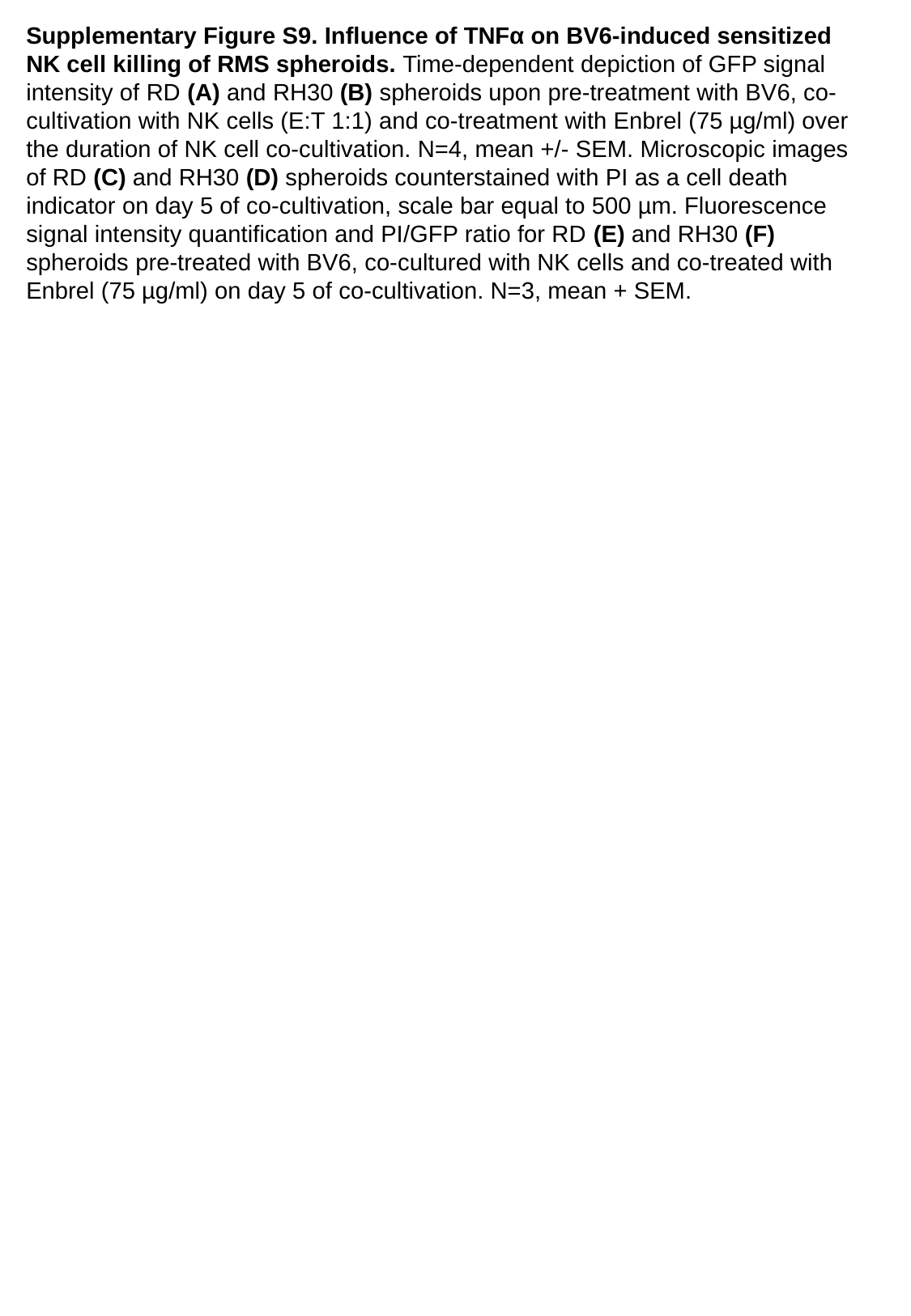

Supplementary Figure S9. Influence of TNFα on BV6-induced sensitized NK cell killing of RMS spheroids. Time-dependent depiction of GFP signal intensity of RD (A) and RH30 (B) spheroids upon pre-treatment with BV6, co-cultivation with NK cells (E:T 1:1) and co-treatment with Enbrel (75 µg/ml) over the duration of NK cell co-cultivation. N=4, mean +/- SEM. Microscopic images of RD (C) and RH30 (D) spheroids counterstained with PI as a cell death indicator on day 5 of co-cultivation, scale bar equal to 500 µm. Fluorescence signal intensity quantification and PI/GFP ratio for RD (E) and RH30 (F) spheroids pre-treated with BV6, co-cultured with NK cells and co-treated with Enbrel (75 µg/ml) on day 5 of co-cultivation. N=3, mean + SEM.
